# Prevalence of Anxiety in Medical Students during the COVID-19 Pandemic: A Rapid Systematic Review with Meta-Analysis

**DOI:** 10.3390/ijerph17186603

**Published:** 2020-09-10

**Authors:** Isabel Lasheras, Patricia Gracia-García, Darren M. Lipnicki, Juan Bueno-Notivol, Raúl López-Antón, Concepción de la Cámara, Antonio Lobo, Javier Santabárbara

**Affiliations:** 1 Department of Microbiology, Pediatrics, Radiology and Public Health, Faculty of Medicine, University of Zaragoza, Building A, 50009 Zaragoza, Spain; isabel.lasheras@hotmail.es (I.L.); jsantabarbara@unizar.es (J.S.); 2 Psychiatry Service, Hospital Universitario Miguel Servet, Paseo Isabel la Católica, 1-3, 50009 Zaragoza, Spain; pgraciag@salud.aragon.es; 3 Centre for Healthy Brain Ageing, School of Psychiatry, University of New South Wales Medicine, Randwick 2052, Australia; d.lipnicki@unsw.edu.au; 4 Centro de Investigación Biomédica en Red de Salud Mental (CIBERSAM), Ministry of Science and Innovation, Avenue Monforte de Lemos, 3-5, Pavilion 11, Floor 0, 28029 Madrid, Spain; rlanton@unizar.es (R.L.-A.); conchidlc@hotmail.com (C.d.l.C.); 5 Instituto de Investigación Sanitaria de Aragón (IIS Aragón), Zaragoza, Spain, Avenue San Juan Bosco, 13, 50009 Zaragoza, Spain; alobo@unizar.es; 6 Department of Psychology and Sociology, Universidad de Zaragoza, Pedro Cerbuna, 12, 50009 Zaragoza, Spain

**Keywords:** anxiety, COVID-19, prevalence, medical students, meta-analysis

## Abstract

The novel coronavirus disease (COVID-19) pandemic has brought a great deal of pressure for medical students, who typically show elevated anxiety rates. Our aim is to investigate the prevalence of anxiety in medical students during this pandemic. This systematic review and mini meta-analysis has been conducted following the PRISMA guidelines. Two researchers independently searched PubMed on 26 August 2020 for cross-sectional studies on medical students during the COVID-19 outbreak, with no language restrictions applied. We then performed a manual search to detect other potentially eligible investigations. To the 1361 records retrieved in the initial search, 4 more were added by manual search on medRxiv. Finally, eight studies were finally included for qualitative and quantitative analysis, which yielded an estimated prevalence of anxiety of 28% (95% CI: 22–34%), with significant heterogeneity between studies. The prevalence of anxiety in medical students is similar to that prior to the pandemic but correlates with several specific COVID-related stressors. While some preventive and risk factors have been previously identified in a non-pandemic context, knowledge and cognitions on COVID-19 transmission, treatment, prognosis and prevention negatively correlate with anxiety, emerging as a key preventive factor that may provide a rationale for why the levels of anxiety have remained stable in medical students during the pandemic while increasing in their non-medical peers and the general population. Other reasons for the invariability of anxiety rates in this population are discussed. A major limitation of our review is that Chinese students comprised 89% the total sample, which could compromise the external validity of our work

## 1. Introduction

The outbreak of the novel coronavirus disease (COVID-19) in Wuhan, China, in December 2019 has rapidly escalated into a global health crisis and was declared a pandemic by the World Health Organization (WHO) on 11 March 2020 [1]. To date (2 September 2020), there have been 25,602,665 confirmed cases of COVID-19 worldwide and 852,758 deaths [2]. Due to the rising numbers of disease cases and deaths, and the highly contagious nature of the disease, some countries began quarantining their population for indefinite periods of time in order to prevent the spread of the disease [3]. While such restrictive measures can be effective in the containment of the virus, concern has arisen about their possible psychological impact on the well-being of the general population and individuals who might be vulnerable to mental health diseases [4], as anxiety and depression have been demonstrated to stem from similar circumstances in the past [5,6].

Several sources of stressors related to pandemics and their Public Health management have been described in the past, such as the unpredictable nature of the disease [7,8], the lack of timely and transparent information disclosure by authorities [3], the loss of personal freedom, sudden changes and impossibility of future planning and social distancing, together with the worry about one’s own health and that of one’s relatives and acquaintances [8], and the large financial losses expected [9]. A recent systematic review on the psychological impact of previous confinements related to the Ebola, H1N1 influenza, Middle East respiratory syndrome (MERS-CoV) and equine influenza disease outbreaks, found that a long duration of quarantine, fear of infection, inadequate information, stigma, or financial loss were related to higher levels of anxiety, anger, confusion, and post-traumatic stress [10]. This is underpinned by a recent meta-analysis reporting a prevalence of anxiety of 31.9% among the general population during the COVID-19 pandemic [11].

For medical students specifically, high anxiety levels were also found during the previous MERS-CoV and SARS-CoV-1 outbreaks [12,13]. Medical students are recognized as an at-risk group for developing anxiety disorders [14], with significantly larger rates than the general population, even under normal circumstances, especially for those residing in the Middle East and Asia [14,15]. Besides being less likely to seek support when affected by psychological distress [14], their distinctive personality traits, such as the high proportion of students with maladaptive perfectionism [16], might make them especially sensitive to the distress and disruption of routine caused by COVID-19 and its associated Public Health measures [17]. Moreover, unlike other students, they have a deeper understanding of the disease, which could increase awareness of its severity and impact [18]. Furthermore, they have been subject to different strategies in regard to disease control. While some medical schools have forbidden students from any patient interaction, as recommended by the American Association of Medical Colleges (AAMC), stripping the students of a fundamental part of their curriculum, other students have been recruited for hospital-based roles to counteract the health system saturation [19]. Either way, the psychological consequences of such drastic changes in their education should be addressed.

There have been several reports, opinion articles and studies recently published on the psychological impact of the COVID-19 pandemic on college and medical students specifically. Our goal is to conduct a systematic review and meta-analysis of studies investigating the prevalence of anxiety in medical students during the COVID-19 pandemic.

## 2. Materials and Methods

This meta-analysis follows the PRISMA guidelines for reporting systematic reviews and meta-analysis [20] (Supplementary Materials Table S1).

### 2.1. Search Strategy

Two researchers (J.B.-N. and P.G.-G.) searched for all cross-sectional studies reporting the prevalence of anxiety published until 26 June 2020 using MEDLINE via PubMed. The Pubmed search was: (covid or covid-19 OR coronavirus OR “corona virus” OR SARSCoV-2 OR “Coronavirus”[Mesh] OR “severe acute respiratory syndrome coronavirus 2”[Supplementary Concept] OR “COVID-19”[Supplementary Concept] OR “Coronavirus Infections/epidemiology”[Mesh] OR “Coronavirus Infections/prevention and control”[Mesh] OR “Coronavirus Infections/psychology”[Mesh] OR “Coronavirus Infections/statistics and numerical data”[Mesh]) AND (anxiety OR anxiety symptoms OR anxiety disorders OR anxious OR “Trauma and Stressor Related Disorders”[Mesh] OR “Anxiety”[Mesh] OR “Anxiety Disorders”[Mesh] OR “Anxiety/epidemiology”[Mesh] OR “Anxiety/statistics and numerical data”[Mesh]). No language restriction was made. References from selected articles were also inspected to detect additional potential studies. We then performed a manual search of the “grey literature” (e.g., medRxiv or Google Scholar) to detect other potentially eligible investigations. Any disagreement was resolved by consensus with a third and fourth reviewer (J.S. and I.L.). This search was updated on 26 August 2020.

### 2.2. Selection Criteria

Studies were included if: (1) reporting cross-sectional data on the prevalence of anxiety, or sufficient information to compute this, conducted during the COVID-19 outbreak; (2) focused on medical students; (3) included a validated instrument to assess or diagnose anxiety disorders; (4) the full text was available. We excluded studies focusing on community-based samples or specific samples that were not medical students (e.g., medical professionals, patients), as well as review articles.

A pre-designed data extraction form was used to extract information on the following: country, sample size, prevalent rates of anxiety, proportion of females, average age, instruments used to assess anxiety, response rate, and sampling methods.

### 2.3. Methodological Quality Assessment

Articles selected for retrieval were assessed by two independent reviewers (J.B.-N. and J.S.) for methodological validity before they were included in the review using the Joanna Briggs Institute (J.B.I.) standardized critical appraisal instrument for prevalence studies [21] (Table S2). Quality was evaluated according to nine criteria, each yielding a score of zero or one. One score was obtained for each criterion if the study was affirmative in the next questions: (1): Was the sample frame appropriate to address the target population? (2): Were study participants recruited in an appropriate way? (3): Was the sample size adequate? (4): Were the study subjects and setting described in detail? (5): Was data analysis conducted with sufficient coverage of the identified sample? (6): Were valid methods used for the identification of the condition? (7): Was the condition measured in a standard, reliable way for all participants? (8): Was there appropriate statistical analysis? (9): Was the response rate adequate, and if not, was the low response rate managed appropriately?

Any disagreements that arose between the reviewers were resolved through discussions, or by further discussion with a third reviewer (P.G.-G.).

### 2.4. Data Extraction and Statistical Analysis

Frequency measures (prevalence) for anxiety and the 95% confidence interval (95% CI) were obtained from each included study. A generic inverse variance method with a random effect model was used to estimate pooled prevalence rates [22]. Random effect models are more appropriate than fixed effect models when the number of studies included in the meta-analysis is low (<10) [23]. The Hedges Q statistic was reported to check heterogeneity across studies, with statistical significance set at *p* < 0.10. Following the recommendations for a small number of studies [24], the *I^2^* statistic was also used to quantify heterogeneity. *I*^2^ values between 25% and 50% are considered as low, between 50% and 75% as moderate, and 75% or more as high. Sources of heterogeneity can include differences in study design or in demographic characteristics. We performed meta-regression and subgroup analyses to explore the sources of heterogeneity expected in meta-analyses of observational studies. We conducted a sensitivity analysis to determine the influence of each individual study on the overall result by omitting studies one by one. Publication bias was determined through visual inspection of a funnel plot and Egger test (*p* values < 0.05 indicate publication bias).

J.B.-N., I.L. and J.S. conducted data extraction and the assessment of methodological quality. Periodic meetings were held to minimize the risk of errors at each step of the review process. In the case of disagreement between researchers, they were asked to reach consensus.

Statistical analyses were conducted by J.S. and run with STATA software (version 10.0; College Station, TX, USA).

### 2.5. Data Availability Statement

Data are available to qualified investigators on request to the corresponding author. Data will be shared at the request of other investigators.

## 3. Results

### 3.1. Identification and Selection of Articles

**Figure 1:**
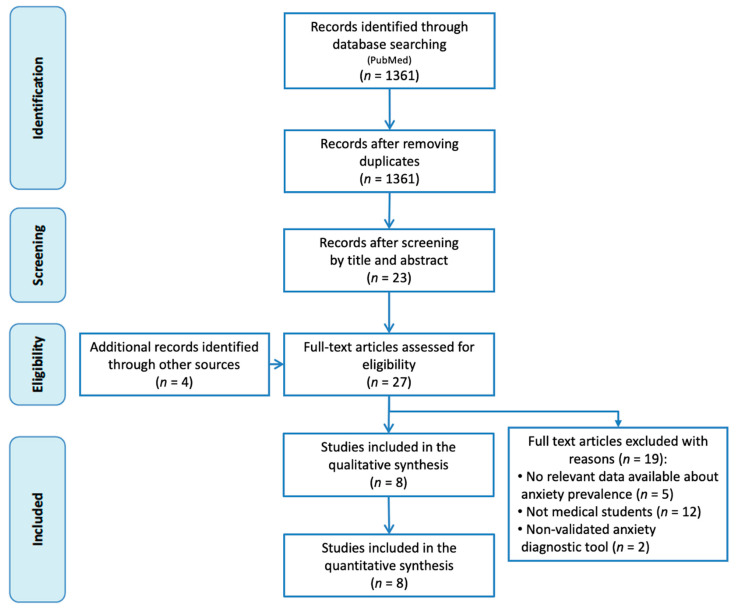
Flowchart of the study selection.

Figure 1 shows a flowchart of the literature search strategy and study selection process. Initially, 1361 potential records were identified, from which 1338 were excluded after the screening of the titles and abstracts for failing to meet the inclusion criteria. To the remaining 23 articles we added 4 more found by the manual search. After reading these 27 articles in full, we included 8 in our meta-analysis.

### 3.2. Characteristics of the Studies Included

Table 1 summarizes the characteristics of the eight included studies. The percentage of women was not reported in one study [25], and was only specified for the totality of the sample (medical and non-medical students) in another one [6], but reached almost 70% of medical students in the largest study of all [26]. Four studies investigated the prevalence of anxiety in students residing in China, specifically from Fujian Medical University (Fujian) [25], Changzhi Medical College (Hubei) [26], Tongji Medical College (Hubei) [18], Capital Medical University (Beijing) and Huazhong University of Science and Technology (Wuhan) [27], whereas the remaining investigated medical students from universities located in the United Arab Emirates [6], Iran [28], Brazil [29], and India [30]. Data were retrieved from February to May, with students being quarantined at the time of the study in all of them. All studies were carried out using an online survey which comprised demographic information (not provided in one case) [25] and the evaluation of anxiety levels with the 7-item Generalized Anxiety Disorder Scale (GAD-7) in five cases [6,18,26,27,29] and the State-Trait Anxiety Inventory (STAI-6) [25], the Beck Anxiety Inventory (BAI) [28], and the Depression Anxiety Stress Scale (DASS-21) [30], in one case.

Additionally, some of them investigated variables related to the students’ sources of information, perceived sufficiency of information and media exposure [6,25], their level of knowledge on COVID-19 statements related to its transmission, treatment, prognosis and prevention [6], their cognitions from the epidemic and preventive response [6,25,26], some COVID-related stressors such as the influence of the epidemic on their economy, academic delays and daily life [26], partaking in high-risk ward clinical rotation, contact with suspected infected patients [6] and having a relative or acquaintance be infected [26]. For instance, Saddik et al. [6] reported a higher median score for knowledge of Covid-19 in medical students than in non-medical students (*p* < 0.0001), as well as a higher perception of knowledge of prognosis and transmission of the virus (*p* < 0.0001). Similarly, protective factors such as availability of social support and living with parents were surveyed in two studies [6,26], and some investigated additional psychological responses to the epidemic, such as feelings of fear, avoidance and embarrassment [6,25] and presence of depression as evidenced by the Patient Health Questionnaire-9 (PHQ-9) [18], Beck Depression Inventory (BDI) [28] or DASS-21 [30].

### 3.3. Quality Assessment

The risk of bias scores ranged from 6 to 9 out of a possible total of 9, with a mean score of 7.6 (Table S2). The most common limitations were: (a) response rate not reported, or large number of non-responders (five studies), and (b) recruitment of participants not appropriate (two studies), and (c) study subjects and setting not described in detail (two studies).

### 3.4. Meta-Analysis of the Prevalence of Anxiety

**Figure 2:**
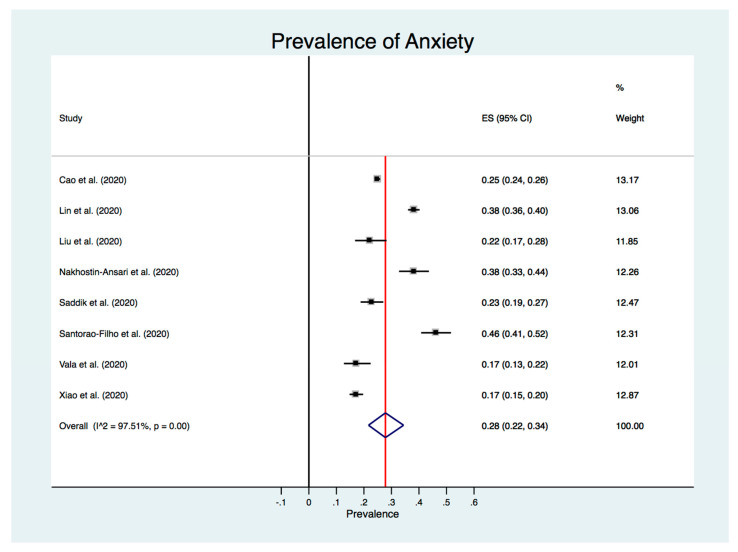
Forest plot.

The estimated overall prevalence of anxiety in medical students during the COVID-19 pandemic was 28% (95% CI: 22–34%), with significant heterogeneity between studies (*I*^2^ = 97.5%, *p* < 0.001) (Figure 2).

### 3.5. Meta-Regression and Subgroup Analysis

The only relevant finding was a slightly lower prevalence of anxiety for the studies using the GAD-7 (26% [95% CI: 19–33%]) [25] compared to those using the STAI-6, BAI or DASS-21 (31% [95% CI: 20–43%]) [6,16,27], and for the studies carried out in China (China: 25% [95% CI: 17–34%] vs. other countries: 30% [95% CI: 18–44%]), according to subgroup analysis. Sampling method (cluster or random sampling: 25% [95% CI: 24–26%] vs. convenience sampling: 27% [95% CI: 17–37%]) was not a moderator. Our meta-regression showed that the prevalence of anxiety was independent of the methodological quality (*p* = 0.725).

### 3.6. Sensitive Analysis

Excluding each study one-by-one from the analysis did not substantially change the pooled prevalence of depression, which varied between 25% (95% CI: 20–32%), with Sartorao-Filho et al. [29] excluded, and 30% (95% CI: 23–37%), with Xiao et al. [27] excluded. This indicates that no single study had a disproportional impact on the overall prevalence.

### 3.7. Publication Bias

Visual inspection of the funnel plot (Figure S1) give the impression of perfect symmetry around the vertical axis, and the results from Egger’s test imply that there was no statistically significant systematic relationship between the results of each study and its size (*p* = 0.722).

## 4. Discussion

Medical students show higher baseline rates of anxiety compared to the general population [10] and their age-matched peers [31]. There are several proposed mechanisms, including a high proportion of students with neurotic and perfectionistic personalities [16,31], and a particularly academically and emotionally demanding training [16,32,33].

Since student distress and untreated anxiety are reported to negatively impact academic performance, professionalism and empathy towards patients, and contribute to academic dishonesty and attrition from medical school [16,31], addressing the effect of COVID-19 on this specific population is of uttermost importance. In addition, the personal costs of anxiety should not be overlooked, since it is associated with a lower quality of life [34], loss of relationships [35] and depression [36], among other things.

We estimate a prevalence of anxiety among medical students during the COVID-19 pandemic of 28%. Sociodemographic correlates varied across studies. For instance, while higher levels of anxiety were found for female Saudi, Brazilian and Iranian students [6,28,29], this difference was only significant in one Chinese study [27]. A higher prevalence of anxiety in women would be consistent with evidence from the prior epidemic of the Middle East Respiratory Syndrome-Corona Virus (MERS-CoV) [37]. With regard to the students’ location, neither Cao et al. [26], nor Liu et al. [18], found any differences in living inside or outside Hubei, the epicenter of the pandemic. On the contrary, Xiao et al. [27] found a significantly higher prevalence of anxiety in students attending university in Wuhan than those in Beijing, a far less severely affected area by COVID-19. Likewise, higher anxiety levels were noted for students living in rural areas, perhaps due to poorer economic conditions and less sanitary resources and preventive strategies [26].

Other stressors identified in the medical student population include worry about the economic influences, academic delays, and the impacts on their daily life [26]. Curricular factors, such as unstructured or online learning, might promote distress and burnout among medical students [31,38], and could be contributing to anxiety. In this sense, two Chinese studies revealed that the impact of online learning appeared to be higher in students from senior years rather than previous-year students, most likely due to a more tightly packed curriculum [4,27]. This hypothesis, however, was not supported by other studies on medical students included in our meta-analysis, since Cao et al. [26] and Liu et al. [18] found no association between grade and anxiety and Lin et al. [25] found a gradual decrease in the proportion of moderate-to-severe anxiety by grades.

In a similar fashion, some other risk factors correlating with the presence and severity of anxiety, such as the unsteadiness or lowness of family income, having COVID-19 symptoms or having a relative or an acquaintance infected with COVID-19 [18,26,28], are not exclusive to the student population, as they have been identified in the general population [5,39]. Nevertheless, the impact of the pandemic on student’s financial ability to continue on course was identified as a major source of anxiety and depressive symptoms in medical students and should be addressed by the authorities [29].

Interestingly, a prevalence of anxiety of 28% is lower than the prevalence prior to COVID-19 for medical students globally, which was estimated as 33.8% in a recent meta-analysis [14], and similar to the baseline rates reported in Chinese students by a systematic review published last year, where the mean prevalence of anxiety was 27.2% [15]. This finding also contrasts with the tendency of anxiety rates in the general population, where it could have increased by four-fold [11]. Moreover, further differences were found by two studies that compared medical students to their non-medical peers during the confinement: one of them found medical students to be less likely than non-medical students to suffer from moderate anxiety [40] and the other one found lower anxiety levels in comparison to dental medicine students [6].

This could be explained by several reasons. First of all, medical students were found to have a higher perceived sufficiency of information on COVID’s prognosis and transmission, and a broader knowledge of the disease compared to their counterparts, perhaps due to a significantly higher use of official sources of information (WHO website, press releases from the Ministry of Health and hospital announcements) [6], which could contribute in turn to a reduction in their fears and anxiety [6,41]. This has already been shown in medical staff facing previous health crises, where perceived sufficiency of information about the A/H1N1 influenza prognosis was independently associated with reduced degree of worry [42]. While the correlation of COVID-19 knowledge and anxiety did not reach statistical significance in another study conducted on midwifery students [43], it showed contribution to lowering perceived levels of stress, which do correlate with anxiety [26]. This reinforces how timely and transparent information, which is critical for healthy psychological self-adaptation regarding fast onset emergencies [3], might not have been accurately delivered to the general population, who could have been more exposed to sensational misinforming news reports in unofficial channels, whereas medicine students could have been prematurely aware of a belated official information disclosure [3,44]. For instance, a study on Wuhan’s university population claimed that many students were aware of the existence of a respiratory disease before the release of the first government notice on 30 December 2019 [3].

Secondly, medical students show high levels of resilience, which positively correlates with adaptive coping strategies when facing a problem [45], and has been shown to prevent the development of anxiety, as well as post-traumatic stress disorder and depression [46].

Thirdly, since many of the reasons for baseline high levels of anxiety in medicine students are academic-related, it is possible that online learning might have eased the burden of over-loaded academic programs. In fact, one study revealed that 87% of students perceived less income knowledge from online classes and over half of the students were totally satisfied with it [29], and, in another study, anxiety levels significantly decreased and knowledge score stopped being a predictor for medical students’ anxiety after switching to the online learning, in contrast with their non-medical peers [6]. Another reason for this could be that minimization of medical students’ presence in hospitals might have helped control their anxiety symptoms due to being distant from the perceived risk of COVID-19 [6]. This hypothesis is supported by Nakhostin-Ansari et al. [28] and underpinned by the findings of the highest levels of anxiety in medical students who continued their high-risk ward rotations during the pandemic [6]. This is in line with some studies conducted on frontline health care workers, where those engaged in direct diagnosis, treatment, and care of patients with COVID-19 show a higher risk of depression, anxiety, insomnia and distress [47,48].

Moreover, it is also possible that a higher degree of knowledge of the disease could have a positive impact on the students’ preventive behavioral response to the epidemic, boosting a feeling of safeness, since, in one study, medical students showed a greater compliance with avoidance of contact with symptomatic people, as well as a decrease in social visits, attendance of crowded places and use of public facilities [6]. This finding, however, did not reach statistical significance, perhaps mitigated by the positive association of anxiety and compliance with hygienic practices.

Finally, home confinement can bring opportunities for family cohesion and increase the availability of support for medical students who might otherwise struggle to seek it. In fact, living with parents and social support were found to be protective factors for anxiety, along with living in urban areas and family income stability [6,17,26]. Co-residence with parents is common in Chinese society, driven not only by highly resilient traditional values but also by more modern models and needs [49]. We believe it would be beneficial to further study the effect of the pandemic on family cohesion and its relationship with anxiety levels, for which a newly-developed tool could be used [50].

Nevertheless, it should not be overlooked that lockdown may prevent students from engaging in other beneficial activities such as exercise [51,52], which, together with peer support, has been shown to be the most effective non-pharmacological therapy in the college and university student population [53], and was found to alleviate general negative emotions in college students specifically during the pandemic [54]. Similarly, strict quarantine regulations and movement control may also limit access to counselling services, leading to a worsening of previously established anxiety disorders [17,53,54]. It should also be noted that, although their effect might be strengthened under the current circumstances, some of these stressors and protective and risk factors have been previously identified in medical students in a non-pandemic context [14,15,16,31].

Lastly, our study has several limitations. Firstly, it only includes eight studies, one of which had a much larger sample size than the others. However, some research has shown that meta-analysis of few studies could still provide valid information [55]. Secondly, even though only half of the studies were conducted in China, their larger sample sizes resulted in 89% of students being Chinese, which could restrict the generalization of the results. Nevertheless, while significant baseline differences in anxiety have been noted regarding medical students’ continent of residence [14], our study revealed a small difference between the included Chinese and non-Chinese studies, both of which reported lower mean anxiety levels in medical students compared to baseline reference review studies [14,15]. It is worth noting that the tools used for the evaluation of anxiety in all studies have been previously validated for the populations under study. The Chinese version of the State-Trait Anxiety Inventory used in the study by Lin et al. has been duly validated [55] and the Seven-item General Anxiety Disorder Scale used in the other three studies has also been validated for Chinese [56] and Arabic-speaking populations [57]. However, the assessment of anxiety by self-reported scales rather than clinical interviews might bias prevalence rates, because respondents may not respond truthfully but in a socially acceptable way [58].

## 5. Conclusions

In conclusion, the overall level of anxiety in medicine students does not appear to be increased during the COVID-19 outbreak. We hypothesize that this could be related to a broader or earlier knowledge on the virus, a high level of resilience and healthy coping systems, a reduction in the academic load and an increased availability of support within the family.

Nevertheless, we believe an invariable numeric report of the already-high levels of anxiety in this population should not hinder implementation of specific anxiety-reducing strategies, since the several COVID-related stressors identified in this population could significantly affect their typical behavioral cycle of anxiety, as occurred to a sample of American college students (unknown major) in an ecological study, whose increased levels of anxiety and depression did not return to baseline over the break, as typically observed [51].

Furthermore, the protective effect of knowledge on COVID-19 in the development of anxiety, previously evidenced in healthcare workers highlights the importance of transparent information disclosure during health emergencies.

## Figures and Tables

**Table 1:** Characteristics of included studies in the meta-analysis.

Author (Year)	Country	Females (%)	Sample Size (*n*)	Response Rate (%)	Sampling Method	Anxiety Assessment	Prevalence of Anxiety (%)	Quality Score
Cao et al. (2020) [26]	China	69.65%	7143	100%	Cluster sampling	GAD-7 ^1^	24.9%	9
Lin et al. (2020) [25]	China	Not reported	2086	Not reported	Convenience sampling	STAI-6 ^2^	38.1%	6
Liu et al. (2020) [18]	China	41.5%	217	Not reported	Convenience sampling	GAD-7 ^1^	22.1%	7
Nakhostin-Ansari et al. (2020) [28]	Iran	52.3%	323	64.6%	Random sampling	BAI ^3^	38.1%	8
Saddik et al. (2020) [6]	United Arab Emirates	Not reported	418	Not reported	Convenience sampling	GAD-7 ^1^	22.7%	7
Sartorao-Filho et al. (2020) [29]	Brazil	73.80%	340	97.98%	Convenience sampling	GAD-7 ^1^	46.17%	9
Vala et al. (2020) [30]	India	56%	250	Not reported	Convenience sampling	DASS-21 ^4^	17.20%	6
Xiao et al. (2020) [27]	China	70.1%	933	96.2%	Convenience sampling	GAD-7 ^1^	17.1%	9

^1^ GAD-7: Seven-item General Anxiety Disorder Scale. ^2^ STAI-6: Six-item State-Trait Anxiety Inventory. ^3^ BAI: Beck Anxiety Inventory. ^4^ DASS-21: Twenty one-item Depression Anxiety Stress Scale.

## Supplementary Materials

The following are available online at https://www.mdpi.com/1660-4601/17/18/6603/s1, Table S1: PRISMA Checklist, Table S2: Risk of bias assessment, Figure S1: Funnel plot, Reference [8] is cited in the supplementary materials.
