# Erratum: Lasheras, I.; et al. Prevalence of Anxiety in Medical Students during the COVID-19 Pandemic: A Rapid Systematic Review with Meta-Analysis. *Int. J. Environ. Res. Public Health* 2020, *17*, 6603

**DOI:** 10.3390/ijerph17249353

**Published:** 2020-12-14

**Authors:** Isabel Lasheras, Patricia Gracia-García, Darren M. Lipnicki, Juan Bueno-Notivol, Raúl López-Antón, Concepción de la Cámara, Antonio Lobo, Javier Santabárbara

**Affiliations:** 1 Department of Microbiology, Pediatrics, Radiology and Public Health, Faculty of Medicine, University of Zaragoza, Building A, 50009 Zaragoza, Spain; isabel.lasheras@hotmail.es (I.L.); jsantabarbara@unizar.es (J.S.); 2 Psychiatry Service, Hospital Universitario Miguel Servet, Paseo Isabel la Católica, 1-3, 50009 Zaragoza, Spain; pgraciag@salud.aragon.es; 3 Centre for Healthy Brain Ageing, School of Psychiatry, University of New South Wales Medicine, Randwick 2052, Australia; d.lipnicki@unsw.edu.au; 4 Centro de Investigación Biomédica en Red de Salud Mental (CIBERSAM), Ministry of Science and Innovation, Avenue Monforte de Lemos, 3-5, Pavilion 11, Floor 0, 28029 Madrid, Spain; rlanton@unizar.es (R.L.-A.); conchidlc@hotmail.com (C.d.l.C.); 5 Instituto de Investigación Sanitaria de Aragón (IIS Aragón), Avenue San Juan Bosco, 13, 50009 Zaragoza, Spain; alobo@unizar.es; 6 Department of Psychology and Sociology, Universidad de Zaragoza, Pedro Cerbuna, 12, 50009 Zaragoza, Spain

The source of funding was not included in our original article [1]. Therefore, in the section “Funding”, where it is stated: “This research received no external funding”, we would like to add the following information:
